# Cost-effectiveness of community vegetable gardens for people living with HIV in Zimbabwe

**DOI:** 10.1186/1478-7547-12-11

**Published:** 2014-04-30

**Authors:** Chloe Puett, Cécile Salpéteur, Elisabeth Lacroix, Simbarashe Dennis Zimunya, Anne-Dominique Israël, Myriam Aït-Aïssa

**Affiliations:** 1 Action Against Hunger, 247 West 37th Street, New York, NY 10018, USA; 2 Action contre la Faim - France, 4 rue Niepce, 75 662 Paris Cedex 14, France; 3 Action contre la Faim - France, 78-D Thanlwin Road, Bahan Township, Yangon, Myanmar; 4 Action contre la Faim - France, 4356 Cnr Chibuku/Unity Road, Masvingo, Zimbabwe

**Keywords:** Vegetable gardens, Livelihoods, People living with HIV, Food consumption score, Household dietary diversity score, Cost-effectiveness, Societal costs, Mixed methods, Activity-based cost analysis, Zimbabwe

## Abstract

**Background:**

There is little evidence to date of the potential impact of vegetable gardens on people living with HIV (PLHIV), who often suffer from social and economic losses due to the disease. From 2008 through 2011, Action Contre la Faim France (ACF) implemented a project in Chipinge District, eastern Zimbabwe, providing low-input vegetable gardens (LIGs) to households of PLHIV. Program partners included Médecins du Monde, which provided medical support, and Zimbabwe's Agricultural Extension Service, which supported vegetable cultivation. A survey conducted at the end of the program found LIG participants to have higher Food Consumption Scores (FCS) and Household Dietary Diversity Scores (HDDS) relative to comparator households of PLHIV receiving other support programs. This study assessed the incremental cost-effectiveness of LIGs to improve FCS and HDDS of PLHIV compared to other support programs.

**Methods:**

This analysis used an activity-based cost model, and combined ACF accounting data with estimates of partner and beneficiary costs derived using an ingredients approach to build an estimate of total program resource use. A societal perspective was adopted to encompass costs to beneficiary households, including their opportunity costs and an estimate of their income earned from vegetable sales. Qualitative methods were used to assess program benefits to beneficiary households. Effectiveness data was taken from a previously-conducted survey.

**Results:**

Providing LIGs to PLHIV cost an additional 8,299 EUR per household with adequate FCS and 12,456 EUR per household with HDDS in the upper tertile, relative to comparator households of PLHIV receiving other support programs. Beneficiaries cited multiple tangible and intangible benefits from LIGs, and over 80% of gardens observed were still functioning more than one year after the program had finished.

**Conclusions:**

Cost outcomes were 20–30 times Zimbabwe's per capita GDP, and unlikely to be affordable within government services. This analysis concludes that LIGs are not cost-effective or affordable relative to other interventions for improving health and nutrition status of PLHIV. Nonetheless, given the myriad benefits acquired by participant households, such programs hold important potential to improve quality of life and reduce stigma against PLHIV.

## Introduction

Nutrition status is an important factor in the etiology and progression of HIV. Nutrition and immunity are linked, with impaired immune function from suboptimal nutrition status hastening progression of HIV [1]. People living with HIV (PLHIV) have increased nutritional needs, due to malabsorption of nutrients, parasitic infection, and increased resting energy expenditure, among others [2-4]. Poor nutrition status is an independent predictor of mortality for PLHIV undergoing antiretroviral therapy (ART), and adequate diet has been shown to improve adherence to ART [5,6]. Poor diet quality has also been linked with disease severity and mortality outcomes among PLHIV [7].

Food insecurity exacerbates nutritional deficits, and leads to risky coping mechanisms and poor HIV outcomes in resource-limited settings [8-10]. Additionally, PLHIV often suffer from social and economic losses due to the costs of the disease and need support in terms of income generating activities and social empowerment.

Interventions aiming to improve food access and diet quality of PLHIV often focus on provision of food supplements [11]. However food supplementation is unsustainable and does not address the underlying causes of food insecurity [12]. Despite growing interest, there is limited evidence on livelihoods strategies for PLHIV, such as community vegetable gardens. Previous research indicates that livelihoods interventions are well-positioned to reduce stigma against PLHIV by improving their economic status [13]. Evidence of the effectiveness and cost-effectiveness of such strategies would inform decision-making on potential synergies between these and other well-established interventions to support PLHIV, particularly in settings of high HIV prevalence and food insecurity.

Home and community garden projects have demonstrated a broad array of benefits to vulnerable populations in various settings. Improving access to the quality and quantity of food with home gardens helps to improve dietary diversity and micronutrient status [14]. Working together on gardens can provide a source of relaxation and stress relief, increasing feelings of unity among vulnerable communities [15]. Sales of garden produce can supplement household income, thereby augmenting women's contributions to household expenses and increasing their influence on household decision-making; improved income levels have been observed several years after withdrawal of program support, suggesting sustainability of benefits [16].

The limited evidence which exists on home gardens for PLHIV has documented beneficial effects. In Ghana, home gardens were found to contribute significantly to dietary diversity among households of PLHIV, and to be valued as a productive asset by these households [17]. In Cambodia, home gardens brought improvements not only in dietary diversity and household income among PLHIV, but also a reduction in negative attitudes and stigma in both PLHIV and non-PLHIV households [18].

This paper examines an intervention providing home gardens to PLHIV in Zimbabwe, one of the Sub-Saharan African countries most affected by HIV and AIDS, with an estimated prevalence among adults of 13% [19]. From 2007 through 2012, Médecins du Monde (MDM) implemented an intervention to deliver health services to PLHIV in Chipinge District in eastern Zimbabwe, including HIV testing and access to ART via Community-based Counselors (CBCs). Through MDM support groups CBCs provided services to counsel and sensitize the households of PLHIV, including patients, caregivers and dependents, about proper care and treatment for HIV and AIDS, including the benefits of improved nutrition for PLHIV. As part of this intervention, some communities spontaneously developed small nutrition gardens. As demand for these gardens grew, MDM handed over the garden activity to Action Contre la Faim - France (ACF) to scale up.

From January 2008 through January 2011, ACF implemented a EuropeAid-funded program to improve the nutrition and food security status of PLHIV through use of low input gardens (LIGs) in five wards (sub-districts) of Chipinge District. In this rural area, agriculture is the main livelihood activity, and households commonly grow staple crops (maize, millet and sorghum) along with a limited variety of vegetables; production is sub-optimal due in part to high HIV prevalence and poor economic conditions. In order to supplement these local diets, the LIG project was intended to increase and diversify households' home production with a wide variety of nutrient-rich vegetables grown in community gardens. The gardens themselves required minimal, locally-available inputs, and relied on biological control for diseases and pests.

ACF set up gardens, provided fencing, seeds and tools; constructed water points and latrines; and provided training and technical support to beneficiaries. Additional support was provided by Agricultural, Technical and Extension Services (Agritex), Zimbabwe's national agriculture extension program, which acted as a formal partner providing technical support on vegetable cultivation practices to teach households how to achieve maximum yield from the gardens. Participation in the LIG program was voluntary, and beneficiaries comprised PLHIV who were enrolled in MDM support groups, receiving counseling and accessing ART, who also chose to work in the gardens.

At the end of the LIG program, a cross-sectional survey was conducted during an operational research evaluation to assess several outcomes among LIG beneficiaries, including nutritional status (measured as height, weight, body mass index, and mid-upper arm circumference) and several internationally-validated composite measures of both food security (Household Food Insecurity Access Scale) and dietary diversity (measured as Household Dietary Diversity Score: HDDS; and Food Consumption Score: FCS) [20]. Survey results were compared between LIG participant households and “comparator” households of PLHIV registered in MDM support groups and living in the same wards, but not involved in LIGs. All households received other support programs in their communities, such as counseling, testing and access to ART from MDM support groups, and general agricultural extension services from Agritex. The survey found no significant difference between the two groups in terms of nutritional status and food security. However, participants had higher food consumption and dietary diversity scores relative to comparators.

Given these encouraging findings on the effectiveness of LIGs, this analysis has sought to further assess the total cost and cost-effectiveness of LIGs for improving the food consumption (FCS) and dietary diversity (HDDS) of PLHIV, compared to other agricultural and HIV support programs.

## Methods

### Analytical strategy

This study used mixed methods to assess, from a societal perspective, the total cost and cost-effectiveness of LIGs as a strategy for improving the food consumption and dietary diversity of PLHIV in five wards of Chipinge District, relative to comparator households of PLHIV participating in other support programs but not in LIGs. Cost-effectiveness was assessed retrospectively, using accounting records, program documentation, focus group discussions and key informant interviews. All costs are presented in 2010 EUR.

Costs were estimated for the full spectrum of program activities, including all known major interventions received by participant and comparator households which could contribute to food consumption and dietary diversity outcomes. These included other support programs implemented by partner organizations such as MDM, which implemented support groups that provided counseling, testing and access to ART (and upon which the LIG program was based), and Agritex which, in addition to providing specific support to the LIG program on vegetable cultivation practices, also conducted monthly training sessions on general agricultural techniques for communities in the LIG program catchment area, as part of their ongoing extension programs.

Participant households were defined as those taking part in LIG program activities. Aside from the LIG program, the other aforementioned HIV and agricultural support programs were received by both participants and comparators. Comparator households were defined as those receiving only these other support programs, and not LIGs.

ACF accounting data was used to assess total cost of the LIG program. For other support programs, only the costs for activities relevant to diet diversity outcomes were included. These programs were received by both participant and comparator households and therefore costs cancel each other out during incremental analysis. Costs for other support programs were estimated with an ingredients approach, using unit costs and quantities of various inputs [21].

The period of analysis encompasses program implementation from January 2008 to January 2011; gardens were active in only 2 of these 3 years due to political unrest and contracting delays. Additionally, costs of MDM support groups were included from that program's inception in 2007, assuming that sensitization by CBCs about the importance of a healthy diet eventually helped to increase beneficiary acceptance and participation in the LIG program. Beneficiary costs for the program implementation period were estimated during focus group discussions (FGDs), to assess potential benefits and cost savings to households participating in the LIG program. These included both direct and indirect costs (i.e. opportunity costs) of program participation, an estimate of income earned from vegetable sales, and an estimated value of the land donated by beneficiaries and communities for the gardens. Beneficiary perceptions of LIG benefits provide context to quantitative findings.

This analysis employed an activity-based costing (ABC) methodology, wherein program activities were used as an intermediate step to allocate the total cost of a program to its outcomes, rather than only to its inputs as is done with traditional accounting centers (e.g. personnel, equipment, transportation, etc.).

Outcome data for participant and comparator households were taken from the cross-sectional survey conducted at the end of the LIG program, which included measurement of HDDS and FCS [20].

Total cost of the LIG program was divided by number of participant households to estimate cost per beneficiary household. Costs for other support programs were subtracted from cost per beneficiary to estimate incremental cost of the LIG program. Cost-effectiveness was assessed using incremental cost-effectiveness ratios (ICERs). To explore uncertainty in costs and outcomes, we performed univariate and multivariate sensitivity analyses.

### Effectiveness data

Effectiveness data were taken from a cross-sectional survey conducted as part of a separate analysis [20]. For inclusion in the survey, LIG participants were randomly selected from a list of program participants. Given ethical concerns involved in withholding food interventions in this vulnerable population, a randomized controlled trial was not conducted. Instead, the counterfactual was measured in this evaluation using comparator households randomly selected from the lists of PLHIV who were participating in MDM support groups but not involved in LIGs. To account for confounding factors, participants were matched with controls based on their ward of residence. Baseline data were not collected.

The survey found that participant households were different from comparators in that they had received more food assistance, and had more land available for cultivation. Regression analysis was used to control for several variables, including socio-economic status and receipt of food assistance. Higher scores among participant households relative to comparator households for HDDS (6.6 vs. 5.7) and FCS (40.5 vs. 36.1) were found to be independent of these factors.

The HDDS represents a household's dietary diversity, and is measured by summing the number of food groups consumed in the past 7 days, from 12 food groups including cereals, roots and tubers, fruits, fish and seafood, and oils and fats. The HDDS has a minimum score of 0 and a maximum of 12 [22]. The FCS is used in decision-making by the United Nations' World Food Program and represents a household's dietary diversity and food frequency. It is measured by calculating the number of days that a household consumed specific food groups in the past 7 days, multiplying the number of days by a weighted value for each of 9 food groups, and summing across categories to calculate a proxy indicator. An acceptable FCS is defined as being greater than 35 out of a possible 100 [23].

### Data collection

Field data collection for the cost-effectiveness analysis occurred in April and May 2012. FGD guides were revised for clarity with local program staff before beginning data collection.

Institutional costs were estimated via a review of internal documents including program reports and accounting data, along with key informant interviews with relevant staff from ACF and partner organizations. For staff with whom an interview was not possible or practical, time allocation estimates were used from other staff implementing similar activities and from supervisory staff where available. Thirty-three key informant interviews were conducted with current and former ACF staff (n = 25), partner staff (n = 7) and local government officials (n = 1).

To conduct FGDs with households of PLHIV, a list of LIGs was purposively selected to obtain a sample of gardens with variation in source of water, climactic conditions, accessibility, access to markets and available economic opportunities in the surrounding area (e.g. export cropping or ability to grow and sell fruits, etc.). FGDs were conducted at 15 garden sites with participant households (15 FGDs, n = 171 participants total), and 5 sites with comparator households identified via MDM staff (5 FGDs, n = 45 participants total). ACF staff were asked to select beneficiaries at random from the list of garden participants when organizing the discussions. During FGDs, a translator asked questions to community members in the local language of *Shona*, and translated discussions into English.

Participants were asked about time spent working on the gardens, local daily wage rates available for agricultural labor and expenses paid with garden earnings. Comparators were asked about time spent in agricultural training sessions provided in these communities, and wages for common livelihoods in their area.

### Cost estimates and assumptions

Total cost was estimated using a combination of accounting data (for ACF costs) and ingredient cost estimates (for costs to partner organizations and participating households). To estimate partner and beneficiary costs, a micro-costing approach was applied wherein all activities were broken down into their component ingredients, and costs were then estimated for each ingredient [21]. Ingredients included the value of time spent by program participants and institutional personnel, direct costs for training sessions, and an estimate of beneficiary income from the program, which was treated as a negative cost (or financial benefit) and subtracted from other costs during data analysis.

Several assumptions were made in estimating costs. A shadow wage for community members (including LIG beneficiaries and volunteer MDM staff) was estimated using the median wage rate for rural livelihoods in Chipinge from community discussions, at 1 USD per day or 0.20 USD per hour. Within the LIG program, a smaller pilot project was implemented to assess the ability of PLHIV in Chipinge to implement Conservation Farming (CF) practices. Costs and activities relevant to this project were allocated to a separate cost center. Costs of staff time from ACF headquarters were not included in this analysis. See the Additional file 1 for further details on costing assumptions and ingredient cost estimates.

### Data analysis

#### Qualitative analysis of LIG benefits

Field notes from FGDs with community members were transcribed into Microsoft Word [24] and analyzed for themes. Expenses paid by beneficiaries from garden-related earnings were tabulated to aid in estimating approximate income from vegetable sales.

#### Adjusting cost data

Cost data was adjusted in several steps before arriving at the final estimates. Some costs were excluded if they were not determined to contribute to routine program operations nor to HDDS and FCS outcomes achieved. Costs of program assessments were excluded; but costs of routine monitoring were included as these contributed to program effectiveness. Capital items such as computers and cars were amortized using standard tables (3 years for computers, 5 years for cars and other equipment, 10 years for communal land) and discounted at a rate of 3%. Program accounting data was converted to Euros from other currencies on a monthly basis using exchange rates from the Reserve Bank of Zimbabwe. All accounting data and ingredient cost estimates from 2008 and 2009 were adjusted for inflation using a Consumer Price Index [25], and all cost data are presented in 2010 Euros using the December 2010 exchange rate (1 USD = 0.75455 EUR) [26].

#### Deriving activity-based cost centers

After adjustments, cost data was allocated to program activities via the ABC methodology. This was done by direct utilization where possible. Program support costs that were shared among multiple activities (e.g. salaries of management staff) were allocated using time allocation proportions of implementing staff, according to ABC methodology.

To employ the ABC methodology, key informant interviews were conducted with program staff to determine the main program activities and their time dedicated to each activity. For each main program activity, time allocation represents a weighted average of different personnel in different sectors (i.e. Water Sanitation and Hygiene, Food Security) spending varying amounts of time on the activities.

#### Cost analysis

Ingredient costs were estimated using Microsoft Excel software [27]. Costs were assessed both in terms of standard accounting centers based on program inputs, and activity-based cost centers based on cost per activity. To calculate net program costs, an estimate of beneficiary income from vegetable sales was deducted from total costs.

#### Cost-effectiveness analysis

Base case cost-effectiveness results used average cost per beneficiary household and observed HDDS and FCS outcomes among participant and comparator households. Incremental cost effectiveness ratios (ICER) were calculated for both HDDS and FCS outcomes, representing the additional cost of the LIG program for each additional household with HDDS in the upper tertile and with an "acceptable" FCS (>35), relative to other support programs.

##### Sensitivity analyses

Sensitivity analyses were conducted to determine whether base case cost-effectiveness estimates (i.e. ICERs) would change substantially given a plausible level of variation in costs and outcomes. Uncertainty was modeled using TreeAge Pro 2012 software [28], employing a decision tree with two branches (1: LIG + Other support programs; and 2: Other support programs alone) and three main variables: incremental cost per household of the LIG program, and outcomes among participant and comparator households. The model assessed the probability that participant households would achieve an improved HDDS or FCS outcome given a range of plausible costs, and assuming various levels of "willingness to pay" to achieve these outcomes. Willingness to pay represents the value of the ICER (i.e. the incremental cost per additional unit of effectiveness) that ACF, donors, policy-makers, or society at large finds acceptable to achieve a particular outcome, based on the value of that outcome and the funds available to achieve it.

Univariate sensitivity analyses considered two main variables for the LIG program: cost per beneficiary household and outcome achieved (individually for HDDS and FCS). The base case estimates for probability of achieving an improved HDDS or FCS outcome were taken from the LIG survey [20]. During sensitivity analyses, best and worst case scenarios were modeled with a range of +/- 25% on these base case observations. Each parameter was varied one at a time using the best and worst case estimates, and resulting changes in the ICER were summarized in tornado diagrams.

A multivariate probabilistic sensitivity analysis was conducted to assess variation in multiple variables simultaneously, based on 100,000 random "Monte Carlo" simulations. The results were used to create cost-effectiveness acceptability curves for each outcome.

## Results

Results are presented in several stages. First we present a qualitative analysis of beneficiary perspectives on the benefits and costs of the program. Second, an estimate of beneficiary income from vegetable sales is presented. This is followed by an analysis of program costs. Finally, results from the cost-effectiveness analysis are presented.

### Qualitative analysis of LIG benefits to beneficiaries

During community discussions, beneficiaries cited multiple positive effects of the gardens on their households and communities.

#### General benefits

According to LIG members, the gardens provided body-building foods which kept them and their households healthy and strong:

“We don't want to hear people call us 'sick people'; we want to be called 'farmers' because we're healthy”.

“If you had seen us before, you would feel pity because we were small and sick. Now, from eating the garden food, we've grown so healthy and big, you can't even tell we're living with HIV”.

Working among others in the garden gave them a social outlet and relieved isolation and anxiety:

“When the garden started, most of us were very sick and we would think a lot. But now we work in the garden and talk to each other, it relieves our stress”.

“The garden reduced our stress in a way. Now we grow vegetables and sell them, and then use the vegetables for transport costs to get to Chipinge town to get our ARV”.

Beneficiaries also cited other intangible benefits from garden membership, including community cohesion and reduced stigma against PLHIV. Because of their access to a productive asset, they were now in a position to help others:

“At the start of the gardens [in the financial crisis of 2008], people had no money, so we had to do barter trading. We would exchange vegetables for oil. This built a relationship between people in the gardens and the community. There was no more discrimination”.

“People used to discriminate us before. Now that we're working in the garden, we're no longer discriminated. Now people come queuing in the garden asking us for our vegetables”.

Many beneficiaries cited a sense of "unity" with other garden members:

“The garden helped a lot because we developed "one-ness" that was not there before. We don't want to miss each other”.

“The garden gave us unity and one-ness because we are different people from different areas who work in the garden. It also helped us to develop love for each other and we take time to care for garden members who get sick”.

While beneficiaries never spent their own household money on the gardens, many gardens members organized themselves to use proceeds from vegetable sales to pay for upkeep and maintenance of the garden and the water points:

“We are not using our personal funds for the garden, even now [one year after the program ended]. We buy seeds with the money from selling vegetables. When the borehole breaks down, each person needs to sell two bundles of vegetables to buy the part for repair”.

“The person who repaired the borehole charged $10. Garden members contributed $0.50 each and community members also contributed since they use the borehole for water”.

A primary threat to garden sustainability occurred when these upkeep and repair costs exceeded what a community was able to pay:

“We didn't have enough money to finish the latrine, so we didn’t put a roof on it [used thatch instead], no ventilation, no cement. The money from the vegetables was not enough to cover that”.

#### Financial benefits

Beneficiaries also cited specific financial benefits of the program. Many groups started savings schemes where they would loan each other money at interest for large purchases, or during times of trouble. They could sell excess vegetables and buy other groceries, pay for household necessities like school fees for their children, and even afford transportation to Chipinge town to get their antiretroviral drugs (ARV):

“When we have vegetables, each person gets five bundles from their beds and sells them. We give this money to the treasurer. We did that repeatedly until we bought a goat… Our plan is to expand the garden, and buy a fence so that other PLHIV can grow vegetables”.

“When my child was sick, I sold tomatoes for money and managed to take him to different hospitals for medication, and he survived”.

Table 1 presents common expenses paid by beneficiaries with proceeds from garden sales (amounts are in USD). Expenditure costs reported are per household. While many of these represent small expenditures, school fees were an important larger expenditure that beneficiaries strived to pay with garden proceeds. Similarly, others saved to pay for more expensive items like blankets (20–35 USD), cell phones (15–35 USD) and hospital fees and medicines (20–40 USD).

**Table 1:** Beneficiary costs paid from garden proceeds (USD)

**Cost**	**Median**	**Range**	**# FGDs reporting**
School fees			
Primary	10	(5–15)	13
Secondary	45	(35–60)	8
Uniforms			
Primary	6	(5.50-7.50)	7
Secondary	11.50	(10–14.50)	5
Shoes	13.50	(12–15)	2
Stationery			
Blank books	0.30	(0.10-0.50)	6
Ballpoint pens	0.30	(0.20-0.30)	10
Groceries			
Cooking oil (2 L)	4	(4–5)	8
Sugar (2 kg)	2.50	(2.50)	4
Maize (20 L)	5	(5)	4
Animals			
Chicken	5	(5)	9
Goat	20	(15–30)	7
Grinding mill	1	(1)	8
Laundry soap	2	(2)	6
Transport to get ARVs	3	(2–15)	5

### Household income from vegetable sales

Two potential financial benefits of the program were: (1) the expenses paid by households using garden proceeds (which approximates income from vegetable sales), and (2) the cost savings to households in consuming vegetables which they otherwise would have had to purchase. This analysis includes an estimate of the first benefit; an accurate estimate of the second would have involved estimating garden yields, which was challenging due to the time elapsed since implementation, and was beyond the scope of this analysis.

Beneficiary cost data from Table 1 were used to estimate the financial benefit of the LIG program. We estimated an average benefit per household of 91.30 USD per year (for one agricultural season) by summing the median expense of items in Table 1 which were reported in at least half of FGDs (≥7 of 15 total). This amount is similar to findings from a subsequent LIG program implemented by ACF, which showed that LIGs were the main source of household income, with vegetable sales earning households an average of 10 USD per month (range: 3–15), and 85 USD per agricultural season in Zimbabwe [29]. These estimates are conservative in that they do not include cost savings to households in terms of consumption of vegetables which they otherwise would have had to purchase.

Estimated income was converted into 2010 EUR and multiplied by the number of households participating in the project, assuming that only half of the LIGs were active in the first year, for a total of 138,272 EUR. This garden-related beneficiary income was incorporated into the analysis as a financial benefit, by deducting it from program costs.

### Cost outcomes

#### Input cost shares

**Figure 1:**
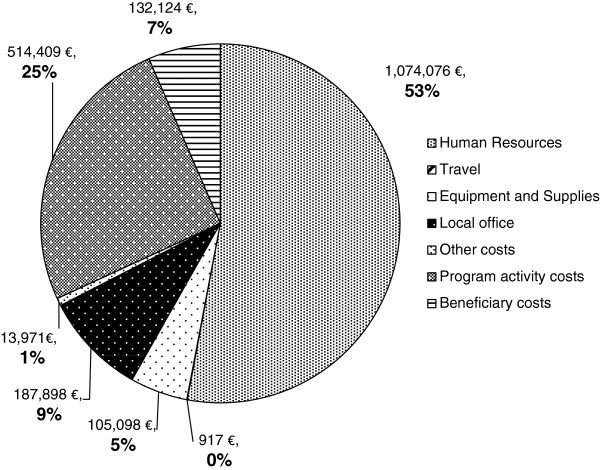
LIG program resource use per input category (EUR).

Figure 1 shows total program resource use by input category. Costs related to program activities represent one quarter (25%) of total resource use and are examined in more detail in the Additional file 2. Human Resource (HR) expenditures represent 53.0% of total program resources, including management and support staff categorized under "support costs". Beneficiary costs comprised 6.5% of total program resource use, and represent net financial costs to the community after subtracting estimated financial benefits in terms of garden-related income. Without deducting garden-related income, beneficiary costs would represent 12.5% of total program resource use. Among the institutions involved, ACF bore the highest proportion of total costs (86.3%), with MDM and Agritex each contributing less than 5% of total costs (4.9% and 2.2% respectively).

#### Activity-based cost analysis

**Figure 2:**
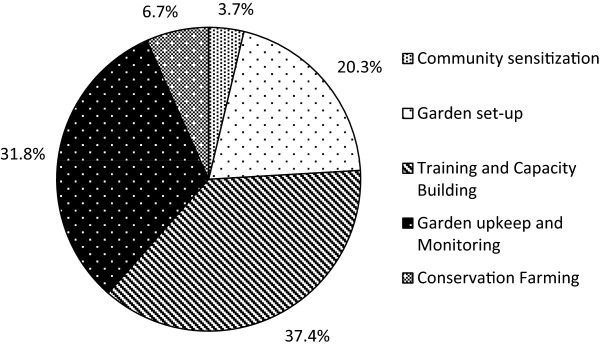
LIG program activity time allocation.

Program activities and staff time allocation proportions derived from the activity-based cost analysis are presented in Figure 2. This figure depicts aggregate time estimates on activities common to all staff, which were used for support cost allocation; time allocation of specific staff on specific activities was used in ingredient cost estimates (Additional file 1). The three activities requiring the most staff time were Training and capacity-building (37.4%), Garden upkeep and monitoring (31.8%) and Garden set-up (20.3%). Table 2 describes activity-based cost centers and data sources for each. Final cost centers included Community sensitization, Garden set-up, Training and capacity-building, Garden upkeep and monitoring and Conservation Farming. To reflect different staff activities and resources used at different points along the program cycle, the activities occurring on the garden sites were split into two activity centers: Garden set-up and Garden upkeep.

**Table 2:** Description of cost centers and data sources

**Cost center**	**Description**	**Data sources**
Community sensitization	Time dedicated to sensitizing community, including local authorities, at beginning of program, by ACF staff and Agritex Extension Officers.	Review of financial documents, time allocation interviews with program and supervisory staff, interviews with Agritex and MDM staff.
	Time dedicated by MDM staff (supervisory staff, field staff and community volunteers) in sensitizing Support Groups.	
Garden set-up	Costs involved in the first months of garden set-up, including costs for constructing gardens and water points, and time dedicated by ACF staff, beneficiaries, and Agritex staff when setting up the gardens.	Review of financial documents, time allocation interviews with LIG beneficiaries, shadow value of garden land as determined by local authorities, time allocation interviews with program and supervisory staff, and interviews with Agritex staff.
Training and capacity building	Costs involved in training both staff and beneficiaries. Cost of beneficiary time in Agritex training sessions on general agricultural techniques (beyond those specifically related to LIGs), time spent by Agritex Extension officers in LIG training sessions and other general agricultural trainings conducted in these communities. Costs of training sessions and refresher training for MDM's CBCs.	Review of financial documents, time allocation interviews with program and supervisory staff, and LIG beneficiaries, and interviews with Agritex and MDM staff.
Garden upkeep and monitoring	Costs involved in upkeep and monitoring of gardens. All beneficiary time spent tending gardens, and an estimate of income from vegetable sales. Agritex Extension Officer time spent monitoring gardens. MDM staff time spent monitoring support groups with gardens (supervisory staff, field staff and community volunteers).	Review of financial documents, time allocation interviews with program and supervisory staff, and LIG beneficiaries, and interviews with Agritex and MDM staff. Focus group discussions with LIG beneficiaries.
Conservation farming	ACF costs to implement and monitor CF activities implemented as a pilot project within the broader LIG program.	Review of financial documents, time allocation interviews with program and supervisory staff.

Table 3 presents total program costs and a comparison of program inputs and costs allocated to each activity-based cost center, both for the LIG program and other support programs received by comparator households. The total cost of the LIG program was 2,028,493 EUR, and the three cost centers requiring the most inputs in the LIG program were Garden set-up (29.1%), Training and capacity-building (33.1%) and Garden upkeep, monitoring and follow-up (26.4%).

**Table 3:** Cost comparison between LIG and other support programs received by comparator households (EUR)

**Cost center inputs**	**LIG**	**Comparator‡**
**Community sensitization**		
*Institutional costs:*		
Personnel	23,668	11,913
Support costs allocated*	37,217	
**Cost center total (% total)**	**60,885 (3.0%)**	**11,913 (8.1%)**
**Garden set-up**		
*Institutional costs:*		
Personnel	50,515	
Travel / transportation	459	
Local office	111	
Other costs, services	3,185	
Program activities	299,289	
Support costs allocated*	202,729	
*Beneficiary costs:*		
Beneficiary time	24,709	
Value of donated land	8,734	
**Cost center total (% total)**	**589,731 (29.1%)**	**-?€-**
**Training and capacity-building**		
*Institutional costs:*		
Personnel	110,274	10,695
Local office	163	
Program activities	115,060	50,461
Support costs allocated*	373,115	
*Beneficiary costs:*		
Beneficiary time¥	73,542¥	73,542
**Cost center total (% total)**	**672,154 (33.1%)**	**134,698 (91.9%)**
**Garden upkeep, monitoring and follow-up**		
*Institutional costs:*		
Personnel	115,683	
Travel / transportation	459	
Local office	111	
Program activities	77,232	
Support costs allocated*	317,372	
*Beneficiary costs:*		
Beneficiary time	163,412	
Household income from vegetable sales†	-138,272†	
**Cost center total (% total)**	**535,997 (26.4%)**	**-?€-**
**Conservation farming**		
*Institutional costs:*		
Personnel	17,494	
Program activities	85,773	
Support costs allocated*	66,459	
**Cost center total (% total)**	**169,726 (8.4%)**	**-?€-**
**Total cost**	**2,028,493 €**	**146,611 €**
Institutional cost	1,896,368 €	73,069 €
Beneficiary cost	132,125 €	73,542 €

**‡**Costs in this column represent other support programs received by comparator households, who did not participate in the LIG program. These additional HIV and agricultural support programs were received by all households (both LIG participants and comparators) and therefore represent common costs between both groups.

*Support costs include cost of program management and support which are shared among program activities. These costs came from ACF accounting and so are assigned to the group receiving the LIG program, which ACF implemented. These costs were allocated to each program activity using an activity-based costing methodology.

¥This line represents beneficiary time in Agritex extension training sessions on general agricultural techniques; this is not an LIG-specific cost and is common to both LIG participants and comparator households.

†This line represents the financial benefit to participant households from vegetable sales, included as a negative cost in this analysis.

### Cost-effectiveness

#### Base case analysis

Table 4 presents base case cost-effectiveness results. In the base case, the LIG program had an average cost of 1,525 EUR per beneficiary household, and an incremental cost of 1,415 EUR per participant household relative to comparator households receiving other support programs. For each additional household achieving HDDS in the upper tertile, the incremental cost of the LIG program compared to other support programs was 12,456 EUR. The incremental cost of the LIG program for each household that achieved an acceptable FCS was 8,299 EUR, compared to other support programs. Cost-effectiveness outcomes from the institutional perspective (i.e. excluding beneficiary costs), and from the ACF perspective (i.e. including only costs from ACF accounting data) were similar to the societal perspective.

**Table 4:** Base case results

**Outcome**	**LIG**	**Comparator**
**Total cost in both areas (EUR)**	2,028,493	146,611
**Incremental cost of LIG (EUR)**	1,881,882^ǂ^	-?€-
Total societal cost per household (EUR)	1,525	110^¥^
Total institutional cost per household (EUR)	1,426	110^¥^
Total ACF cost per household (EUR)	1,317	-?€-
Probability of HDDS being in upper tertile	44.1%	32.8%
Probability of FCS being "acceptable"	59.1%	42.0%
# Beneficiary households	1,330	1,330^¥^
# LIGs constructed	37	-?€-
*HDDS outcome:*		
Incremental cost per household (EUR)^†^	1,415	-?€-
Incremental effectiveness	0.1136	-?€-
ICER (€/household with HDDS in upper tertile)	12,456	-?€-
*FCS outcome:*		
Incremental cost per household (EUR)^†^	1,415	-?€-
Incremental effectiveness	0.1705	-?€-
ICER (€/household with "acceptable" FCS)	8,299	-?€-

HDDS: Household Dietary Diversity Score; FCS: Food Consumption Score.

^ǂ^Incremental costs represent the difference between total costs for LIG and other support programs.

^¥^Comparator households represent MDM beneficiaries participating in other support programs but not the LIG program. For the purposes of comparative analysis, it was assumed that 1,330 comparator households were participating in the other support programs, to match the number of LIG participant households.

†Incremental costs were estimated from a societal perspective.

#### Sensitivity analyses

**Figure 3:**
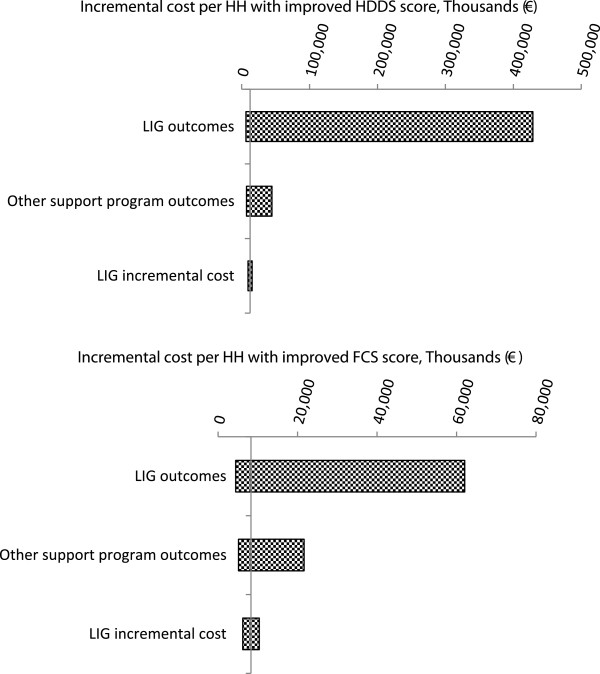
Tornado diagrams for HDDS & FCS outcomes.

Table 5 presents model input parameter values and ranges used in sensitivity analyses. Tornado diagrams for both outcomes (HDDS and FCS) are represented in Figure 3. Variables listed to the left of the diagrams were varied one at a time using the best and worst case estimates from Table 5.

**Table 5:** Model input parameter values and ranges

**Parameter**	**Base case**	**Worst case**	**Best case**	**Source of base case (and range)**
*Effectiveness measures:*				
LIG: Probability of HDDS being in upper tertile	44.1%	33.1%	55.2%	Evaluation data [20]
				*Worst case*: -25% of the base case
				*Best case*: +25% of the base case
Comparator: Probability of HDDS being in upper tertile	32.8%	24.6%	41.0%	Evaluation data [20]
				*Worst case*: -25% of the base case
				*Best case*: +25% of the base case
LIG: Probability of FCS being "acceptable" (>35)	59.1%	44.3%	73.8%	Evaluation data [20]
				*Worst case*: -25% of the base case
				*Best case*: +25% of the base case
Comparator: Probability of FCS being "acceptable" (>35)	42.0%	31.5%	52.5%	Evaluation data [20]
				*Worst case*: -25% of the base case
				*Best case*: +25% of the base case
*Costs per household (EUR):*				
Incremental costs per household in LIG program (societal perspective)	1,415	1,769	1,061	Incremental cost per household (Table 4)
				*Worst case*: +25% of the base case
				*Best case*: -25% of the base case

HDDS: Household Dietary Diversity Score; FCS: Food Consumption Score.

The x-axis represents willingness to pay per household achieving an improved HDDS or FCS score (in thousands of EUR). The y-axis represents the base case ICER estimate for each outcome from Table 4 (12,456 EUR for HDDS; 8,299 EUR for FCS).

For both FCS and HDDS outcomes, one-way sensitivity analyses showed that the model was most sensitive to assumptions about these outcomes (i.e. the probability of participant or comparator households achieving adequate FCS and high HDDS).

**Figure 4:**
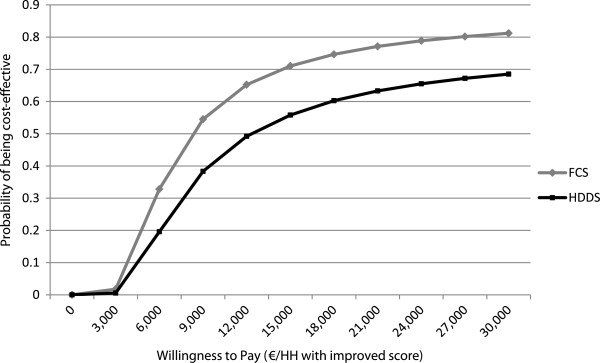
Cost-effectiveness acceptability curves for FCS & HDDS outcomes, from the societal perspective.

Figure 4 presents the results of multivariate sensitivity analyses, in the form of cost-effectiveness acceptability curves for both outcome measurements using total program costs, from a societal perspective. The acceptability curves show that the probabilities that the program would be cost-effective are 50% and 75% at a willingness to pay of 8,376 and 18,426 EUR for improving the Food Consumption Score, and 50% and 65% at 12,353 and 23,308 EUR for improving the Household Dietary Diversity Score.

## Discussion

Providing low input gardens (LIGs) to PLHIV in a 3-year intervention, in partnership with organizations focusing on medical support for PLHIV and agricultural support for rural communities in Zimbabwe, carried an average cost of 1,525 EUR per beneficiary household from a societal perspective. For the money being spent on the LIG program, compared to these other support programs, there was a 40% increase in the number of households with an improved Food Consumption Score (FCS), and a 30% increase in the number of households with an improved Household Dietary Diversity Score (HDDS). The incremental cost for each participant household that had a higher food consumption and dietary diversity score, relative to comparators, was 8,299 EUR and 12,456 EUR respectively. These cost-effectiveness outcomes were sensitive to the assumed level of program effectiveness. The LIG strategy was most likely to be cost-effective if society was willing to pay more than 20,000 EUR to improve dietary diversity and food consumption levels of households of PLHIV. These findings cohere with other qualitative findings suggesting livelihoods programs for PLHIV are generally resource-intensive [12].

We are aware of no other cost-effectiveness studies of similar interventions, or with similar outcomes, with which to compare these results. Cost effectiveness outcomes exist for other HIV-related programming, representing a wide array of interventions, including treatment, prevention and palliative care. Counseling and testing cost 32,000 USD per case of HIV prevented [30]. Reduction of mother to child transmission via breastfeeding and formula-feeding interventions cost between 4,000 USD and 20,000 USD per infection prevented [31]. Prevention of opportunistic infections in patients with advanced HIV cost between 16,000 USD and 314,000 USD per QALY saved [32].

Evidence from food assistance programs provides further context for our findings. In Ecuador, a 7-month food assistance program comparing cash, staple food rations, and food vouchers found a significant increase in HDDS (0.4-0.5 point increase) and FCS (6.1-9.4 point increase) in all 3 interventions relative to a control group [33]. In Niger, households receiving a staple food ration had an average FCS that was 4.6 points higher after 6 months of intervention [34]. Comparing these findings with the difference in scores among participant and comparator households in the LIG program (HDDS: 0.9 points, FCS: 4.4 points) suggests that achievements in this program were comparable with other food assistance programs, but that LIGs may have more potential to increase dietary diversity than food consumption relative to other intervention options.

A food assistance study in neighboring Mozambique found the institutional cost of providing a staple ration to PLHIV to be 288 USD per patient over 3 months [35]. The study in Ecuador found the food ration (delivered to vulnerable households who were not necessarily affected by HIV) to carry the highest marginal cost at 11.50 USD per transfer compared to around 3 USD per transfer for cash and vouchers [33]. Although costing methods differed between these studies and the present analysis and are therefore not directly comparable, these unit cost estimates are up to 2 orders of magnitude lower than those for LIGs.

There is no standard threshold against which to measure these cost-effectiveness outcomes; however Zimbabwe is a poor country with a 2010 per capita GDP of 449 EUR [25]. Considering the broad array of HIV-related interventions that focus on more tangible outcomes like treating and preventing disease, and the high incremental cost of this program for outcomes that are not related to mortality prevention or treating acute illness, other forms of investment may be perceived as more cost-effective by donors or policy-makers [31]. Moreover, given these considerations, this strategy would not necessarily be appropriate for inclusion in essential national packages of health services [36]. Program strategies achieving this designation, such as childhood immunization and insecticide-treated bed nets for example [37], often involve the provision of basic life-saving goods and services, or the treatment of acute illness [38].

Notwithstanding its relative success with dietary diversity outcomes, the high cost to achieve these outcomes lead us to conclude that the LIG program was not cost-effective as a means of improving dietary diversity among PLHIV in this context. While LIGs provide multiple benefits, they are considerably more expensive than other interventions to improve dietary diversity and food consumption.

However, community needs can be complex. As access to ART expands in developing countries, life expectancy for PLHIV will increase. To improve the quality of these life years gained, other more holistic support services, such as income generating activities and other food security interventions will be needed [39-41]. The potential for integrating livelihoods activities into other HIV support mechanisms should be explored, and further evidence is needed to determine which types of livelihoods programs would be best suited for integration [12].

The assets provided by this program - including garden tools, fencing, water points, tool kits, irrigation systems and latrines - contribute to its high costs. The cost of a single borehole can exceed 6,000 USD (ACF accounting records). This is a significant expenditure, although there is a strong humanitarian argument in favor of equitable provision of potable water to vulnerable communities.

A key consideration for cost-effectiveness of LIGs is whether their benefits are sustainable. Over 80% of gardens visited for this assessment were still functioning one year after the program ended, suggesting sustainability of program benefits among the majority of participant communities. Common challenges to garden functioning were soil salinity and malfunctioning water points. The qualitative analysis found that an important threat to garden sustainability was the cost of upkeep and repairs. While garden members can organize to share these expenses, some costs may be beyond communities' ability to sustain in the long-run. This threat to sustainability will need to be considered in future garden interventions.

Previous analyses have documented challenges with participation in public programs if the cost of participation, including the opportunity cost of time spent participating, is too intensive [42-45]. This program required beneficiaries to work in the gardens several days per week. However, given the high levels of continued participation one year after the program ended, it appeared that households assessed the multiple benefits accrued through program participation to outweigh the costs of their time. Moreover, the estimated value of beneficiaries' time in tending the garden (163,412 EUR in Table 3) is nearly balanced out by their estimated income from vegetable sales (138,272 EUR). Taken together, these findings suggest a favorable balance of benefits and costs of program participation, contributing to the continued use of gardens.

Discussions with program staff uncovered inefficiencies and delays in the program timeline, due both to political unrest and delays in contracting construction of latrines and water points. These inefficiencies had implications for program cost-effectiveness by limiting beneficiary time spent in vegetable production, training sessions and other forms of sensitization. This reduced the effective implementation time from 3 to 2 years and may have limited overall effectiveness of the program in improving dietary diversity and food consumption of beneficiary households. Cost-effectiveness of future similar programs could potentially be improved through enhancing efficiency of program activities.

There are important methodological limitations to the effectiveness data on which this cost-effectiveness analysis was based. While the HDDS and FCS outcomes were significant and robust to covariates such as socioeconomic status and receipt of food aid, and although participant and comparator households were matched on ward of residence, we cannot rule out the possibility that participants and comparators may have been different. This is partly due to lack of baseline measurements. Also, due to self-selection of participants, a common challenge when assessing garden programs [46], there may have been other factors affecting diet quality and food consumption than participation in the LIG program itself. Further, given that indexes and controls were taken from the same communities, there is a possibility that the additional training and availability of vegetables from the LIG project may have been disseminated to other community members, including comparator households. This would have made it more difficult to detect an impact of the intervention. In the present analysis, outcome data was subjected to sensitivity analyses, enabling the present study to account for potential variation of +/- 25% in these measurements. Finally, the limitations outlined here would not affect the cost data presented in this analysis.

The cost-effectiveness study was limited in that, at the time of data collection, the program had been completed for over one year. First, this meant that all respondents needed to recall events that happened more than one year in the past. Second, due to the time elapsed we were not able to access all program staff for key informant interviews and to assess their time allocation. Additionally, for the staff with whom we were able to consult, the time elapsed since the end of the program may have made it difficult to recall with accuracy their time expenditure on program activities. However, given that we were able to contact a substantial number of former staff, engaged in a variety of program activities, we do not feel that these potential limitations greatly detract from the usefulness of these findings.

This analysis also has important strengths. While there is growing interest in livelihoods programs for PLHIV, to date there is limited evidence of their effectiveness, and this is the first analysis of which we are aware that assesses cost-effectiveness of such a program. Further, this analysis presents a detailed account of total program costs from a societal perspective, which provides a comprehensive picture of the financial costs and benefits to participating households. Finally, the use of mixed methods enabled us to capture beneficiary perspectives on the myriad tangible and intangible benefits of this program, which help to explain why many households were still working in the gardens more than one year after the program had finished.

### Future research

Previous research has identified a lack of appropriate indicators for livelihoods programs for PLHIV, which capture clinical markers as well as food security status [12]. While cost-effectiveness analysis provides important information on program resource use in monetary terms, it also suffers this lack of comprehensive and appropriate indicators, which future research should strive to address.

Quality of life was assessed in the operational research evaluation for this program, and was not found to differ between participant and comparator households [20]. However, other studies have found important linkages between health-related quality of life and food access and diet quality among PLHIV [47]. This important potential benefit of food assistance and nutrition support programs for PLHIV should be further investigated in other settings.

Additional research should be conducted to quantify the impact of programs like LIGs on other important clinical outcomes, in order to compare the effectiveness and costs of this program strategy more broadly with other HIV-related programming. Further, this analysis has captured costs and benefits during the program cycle; future analyses could measure or model costs and benefits over a longer timeframe to estimate some of the potential longer-term impacts of this program strategy, and whether effectiveness and cost-effectiveness might increase over time.

## Conclusions

Providing low input gardens (LIGs) to PLHIV in rural Zimbabwe in a 3-year intervention, along with agricultural training and sanitation infrastructure, yielded higher food consumption and household dietary diversity outcomes among participant households, relative to comparator households receiving other agricultural and HIV support programs. Communities cited additional psychological, physical, financial and dietary benefits from working in the gardens; moreover many gardens were still functioning one year after withdrawal of program support, suggesting potential for sustainability. However, the cost to achieve these outcomes, at 20–30 times Zimbabwe's per capita GDP, is unlikely to be affordable particularly within government services. Given these findings, this analysis concludes that LIGs are not cost-effective or affordable relative to other interventions for improving health and nutrition status of PLHIV. Nonetheless, given the myriad benefits acquired by participant households, such programs hold important potential to improve quality of life and reduce stigma against PLHIV.

## Competing interests

The authors declare that they have no competing interests.

## Authors’ contributions

CP participated in study design, collected, analyzed and interpreted data and drafted the manuscript. CS participated in study design and coordination, contributed to data interpretation and was involved in drafting the manuscript. EL and SZ contributed to data acquisition and interpretation, and helped to revise the manuscript. MA and AI participated in study design and coordination, and helped to revise the manuscript. All authors read and approved the final manuscript.

## Supplementary Material

Additional file 1 Details on ingredient cost estimates and costing assumptions. Click here for file

Additional file 2 Resource use by program activity. Click here for file
