# Supplementary material for: Cost-effectiveness of community vegetable gardens for people living with HIV in Zimbabwe
Source: Cost Eff Resour Alloc. 2014 Apr 30;12:11. doi: 10.1186/1478-7547-12-11 (PMC4022439; doi:10.1186/1478-7547-12-11)
Supplement: Additional file 1 — Details on ingredient cost estimates and costing assumptions. [file 1478-7547-12-11-S1.docx]

### Additional file 1: Details on ingredient cost estimates and costing assumptions

Assumptions made during cost estimation are listed below, organized by the group incurring the cost.

*ACF costs and assumptions:*

While the LIG program officially began in January 2008, due to the civil and economic unrest in Zimbabwe in mid-2008, program activities were delayed until September/October of that year. Allowing two months for garden setup, a simplifying assumption was made when estimating ingredient costs that beneficiary costs for garden maintenance and Agritex costs for garden monitoring were incurred for only two years (2009-2010).

By the end of the program, 37 gardens had been constructed. A simplifying assumption was made when estimating ingredient costs that half of these 37 LIGs were constructed in the first year (end of 2008) and functioned for two years (2009-2010) and half were constructed in the second year (end of 2009) and functioned for one year (2010). This assumption affects only the ingredient cost estimates for partners and beneficiaries and not the cost of gardens to ACF, which are based on accounting data.

Costs of Conservation Farming (CF) were included in the analysis because the knowledge and practices of some community members in CF were assumed to have a potential effect on the food consumption and dietary diversity of those beneficiary households, with possible spillover effects in the community at large. However as the purpose of this analysis was not to assess the costs of CF, no further costs for these activities were collected beyond the accounting data (including Agritex Officer time specifically supporting CF beneficiaries). Compared to the LIG program, there were relatively few CF beneficiaries (approximately 35 farmers in CF compared to 1,330 households in LIGs), and these overlapped with LIG beneficiaries. Therefore the general costs to beneficiaries of the CF component which would contribute to the functioning of LIGs were assumed to be minimal. Additionally, we assumed that CF practices were not being implemented by comparator households during the time of the program, although they were likely to be adopted after the program ended, as the community's knowledge of these practices spread.

Agritex costs and assumptions:

Agritex implemented other field activities aside from those for the LIG program. Based on information from discussions with participant and comparator households, a simplifying assumption was made when estimating ingredient costs that Agritex activities beyond those related to the LIG program mainly consisted of field-based trainings on general agricultural techniques (occurring monthly or less frequently), and did not include additional monitoring of field cropping practices. Costs to Agritex were included for both the regular monthly trainings they conducted, and for the support they provided as formal partners in the LIG program. The cost of Agritex Extension Officers’ (AOs) time was included from the beginning of ACF program implementation in late 2008.

There was no formal estimate of the number of AOs that worked on the LIG program. Based on key informant interviews with 3 AOs, it was known that some had 1 LIG in their catchment area, and some had 2 LIGs.

Documentation from the beginning of the program planned for 35 AOs. However there are only 14 AOs with over 20 entries (for reimbursements and allowances) in the ACF accounting data. We assumed that these 14 AOs had >1 garden (2 for the purpose of this analysis). Since there are 37 LIGs total, that would leave 9 remaining with 1 garden in their catchment area. Therefore 23 Agritex Officers were assumed to have worked on the program (14+9).

Since implementation began in September 2008, we assumed that AOs had 29 working months in the program, from September 2008 through January 2011.

Based on key informant interviews, it was determined that garden set-up was more time-intensive for AOs since they helped the community plan the garden, clear the fields, peg the beds, and generally set up their garden. This time was estimated to be 60% of their time for 2 months. Thereafter, AOs estimated that they spent between 10% and 12% of their time (11% on average) in routine monitoring of the gardens.

All gardens were not set up at the same time. Therefore it was assumed that half of the gardens in each AO’s catchment area started during the first year of implementation (2008-9) and the second half started in the second year of implementation (2009-10).

For the AOs with 1 garden, this equals to 2 months at 60% time allocation while they were setting up their first gardens, and then 12 months of time at 11% for monitoring these gardens. Then at the beginning of the second year, there were 2 months of 71% time while they are setting up their second gardens (at 60% time) and monitoring other gardens (at 11% time) simultaneously (60%+11%=71%). Assumptions for AOs with 2 gardens are similar, since they were assumed to set up their second gardens in the second year. We assumed the same general amount of monitoring time (11% on average) for both AOs with 1 and 2 gardens since some of them had to travel far to reach their gardens so it may have taken more or less time on average.

The average hourly wage was calculated based on their self-reported (and triangulated) average monthly wage. The sum of these costs of the AOs time was then adjusted for inflation.

Since AOs worked alongside the ACF Agricultural Trainers, we assumed the same activity-based time allocation proportions as were reported by the Agricultural Trainers. These proportions were applied to total calculated ingredients costs to get the proportion of Agritex costs for the various cost centers.

For the Training and Capacity-building cost center, an additional estimate was made for the time AOs spent conducting trainings on general agricultural techniques, assuming a half day (including travel time) once a month for 10 months a year (assuming they did not occur every month of the year), over the course of the whole 3-year program.

MDM costs and assumptions:

MDM's volunteer Community Based Counselors (CBCs) monitored LIGs and provided encouragement and support to communities engaging in nutrition gardening practices. MDM costs were included for the community-based staff who implemented the program (in terms of their trainings, supervisory support, and time spent monitoring and sensitizing communities). Costs for the MDM program were included from the program's inception in 2007, under the assumption that MDM sensitization occurring prior to the beginning of the ACF program in 2008 eventually helped increase beneficiary acceptance and participation in the program, and increased their knowledge about the importance of a healthy diet for PLHIV.

Two main costs were estimated for MDM activities: training and time personnel spent monitoring and sensitizing PLHIV. These cost estimates were derived from key informant interviews with MDM Community Officers, Medical Officers and Administrative Officers.

Training: Training costs were estimated for the initial 5-day CBC training and the subsequent 2 annual 3-day refresher trainings, using costs from MDM for hotel, food allowances, transportation and venue rental.

Sensitization and Monitoring: Personnel time was estimated for 3 Community Officers (COs) and 1 Supervisor, who all oversaw CBCs. These staff estimated that in the wards where the 3 COs in the LIG program area worked, they allocated 30% of their time to the Support Groups (SGs) that had LIGs. Staff mentioned that this time was evenly divided between monitoring and sensitizing these communities about health and nutrition-related information for PLHIV.

The following calculations were made to arrive at the # of CBCs involved in the ACF program:

37 LIGs

1 CBC per Support Group (SG)

15 beneficiaries per SG

3 SGs per LIG (15 beneficiaries per SG, 45 beneficiaries per LIG)

1 CBC per SG, 3 SGs per LIG, 3 CBCs per LIG.

3 CBCs x 37 LIGs = 111 CBCs involved in the program.

Each CBC was assumed to monitor each SG once per month for the full 4 years of the MDM program (2007-2010). This was calculated as 1 day per month at a shadow daily wage of 1 USD (based on opportunity cost of time as calculated for LIG beneficiaries), and assuming it took a CBC one day to make a monitoring trip to a SG, including travel time. Half of this CBC monitoring time was allocated to the "monitoring" cost center, the other half to the "sensitization" cost center since they reported that their time was split more or less evenly between these activities during such visits.

For comparator households, costs were estimated for CBC trainings. Half the estimated staff monitoring time (including CBC time, along with a portion of the CO and supervisor time) was allocated to the sensitization cost center, but no costs were allocated for monitoring since these households would only benefit from general sensitization and not sensitization during monitoring of gardens.

Beneficiary costs and assumptions:

Beneficiary opportunity costs were estimated for time spent working on LIGs, and for time spent in Agritex trainings on general agricultural techniques. Beneficiary time spent on field agriculture was not included as it was determined to be similar between participant and comparator households, thus not contributing to differential outcomes. Beneficiary opportunity costs were not estimated for time spent specifically in the MDM program. All beneficiary time spent in the gardens is included in the Garden set-up and Garden upkeep cost centers. Beneficiary costs in the Training cost center relate only to Agritex trainings, not to trainings received for LIGs. This is because the trainings they received for the LIG program often blended into the ongoing monitoring and follow-up support received from ACF and Agritex staff.

Methods for estimating beneficiary income from garden-related sales are detailed in the article. Calculations of beneficiary income used the same assumptions on garden functionality as above: that gardens were only functional during two years of the program, with half of the gardens functioning in the first year, and with all gardens functioning in the second year.

As mentioned in the article, the shadow wage estimate for rural livelihoods in Chipinge, was 1 USD per day or 0.20 USD per hour. This estimate, obtained from PLHIV during FGDs, was lower than that for workers who were not HIV+. This was because higher-paid work typically involved more intensive manual labor, for which PLHIV did not always have adequate strength. Although the Zimbabwean economy was failing in 2008 at the beginning of the program, an assumption was made when estimating ingredient costs that all beneficiary households would have access to work at this lower wage of 1 USD per day throughout the course of the program.

Beneficiaries in 15 different garden sites reported their average time allocation on various garden- and agriculture-related activities. Using the shadow wage estimated during discussions with LIG beneficiaries, the opportunity cost of beneficiary time was estimated, based on an estimation of their time spent in:

Agritex trainings on general agricultural practices

Working on their gardens:

**Garden set-up**: Beneficiaries estimated the amount of time spent at the beginning of the program, to prepare the land for their garden site. On average they estimated that this activity took four weeks, and that they worked six days per week intensively to set up their gardens. Beneficiaries had trouble remembering the exact number of hours per day they had spent setting up their gardens at the beginning of the program. Therefore when estimating the value of their time, the daily shadow wage rate was used rather than the hourly rate. As mentioned previously, one-half of the gardens were assumed to have started in the first year of implementation (2008-9), and the second half started in the second year of implementation. These costs were adjusted for inflation and are presented in 2010 EUR.

**Garden upkeep**: Beneficiaries reported the days per week and hours per day they would work on their gardens during different seasons of the year. During the summer months, they reported lower time allocation on the gardens since they were focusing on tending their own field crops. During winter months, when the field crops were not growing, they were able to tend to LIG plots for more hours per day. However, throughout the year and regardless of season, beneficiaries reported visiting LIG plots 3 days per week.

The table below presents average beneficiary time allocation on LIGs per month:

| **Month(s)** | **Hours per day** |
| --- | --- |
| September-March | 2.0 |
| April | 3.0 |
| May | 3.5 |
| June | 4.0 |
| July | 2.5 |
| August | 2.5 |

In addition to this time at the garden, beneficiary travel time to the garden was also measured. Beneficiaries reported traveling (by foot) an average of 30 minutes’ travel each way, or 1 hour round trip. These estimates were included in the time allocation estimation for the LIGs.

Time allocation estimates were compiled as number of working hours per year, per beneficiary household. These hourly estimates were then multiplied by the hourly shadow wage rate (0.20 USD) to get a total cost of beneficiary time on the garden per year.

As mentioned above, it was assumed that half of the gardens were set up during the first year of implementation (2008-9) and the second half started in the second year of implementation (2009-10). Therefore costs are only estimated for one-half of the total 1,330 participating households for the first year of implementation, while in the final year all of the households are assumed to have participated for the entire year. These costs were adjusted for inflation and are presented in 2010 EUR.
