# Supplementary material for: Cost-effectiveness of community vegetable gardens for people living with HIV in Zimbabwe
Source: Cost Eff Resour Alloc. 2014 Apr 30;12:11. doi: 10.1186/1478-7547-12-11 (PMC4022439; doi:10.1186/1478-7547-12-11)
Supplement: Additional file 2 — Resource use by program activity. [file 1478-7547-12-11-S2.docx]

**Additional file 2: Resource use by program activity**

Expenses dedicated to program activities varied by cost center.

The Community sensitization cost center reflects time and costs spent by ACF and its partners at the beginning of the program, raising awareness among local authorities and communities about the benefits of improved nutrition for PLHIV. These specific activities were undertaken in order to generate interest among community members to enroll in the LIG program, and participate in garden activities, despite the conspicuous public nature of these activities and the widespread stigma against PLHIV which existed at the time. While the costs dedicated to this activity are relatively small compared to the others, in reality there is also a large component of sensitization and awareness-raising involved in the acts of training and monitoring.

Costs to set up the gardens were different from those to maintain them. Garden set-up costs were predominantly (65%) dedicated to Water & Sanitation construction (Figure 1). This included the tool kits used to construct and repair the water points, parts for the wells and boreholes, and fees paid to subcontractors for the construction of new water points and the rehabilitation of existing ones.

Training and capacity building costs (Figure 2) were primarily (55%) dedicated to annual training sessions hosted by MDM for Community-based Counselors (CBCs) who counseled and sensitized Support Group members (comprised of both participant and comparator households). The next-highest proportion of trainings costs (26%) were dedicated to training sessions conducted by ACF for LIG members and "lead farmers" participating in the Conservation Farming pilot activities. Another major cost (17%) was technical assistance, including training of trainers, development of tools and other technical inputs.

Accounting costs allocated to garden upkeep and monitoring (Figure 3) were made up of half the costs of several budget lines which were assumed to be shared between garden set-up and garden upkeep cost centers. These included costs for tools, seeds and the tool kits and spare parts purchased for the upkeep and repair of the water and sanitation components (e.g. latrines and boreholes). These costs would be expected to take on a larger proportion of overall costs if the program continued for a longer period of time, and regular maintenance and repair costs were incurred.

Accounting costs for Conservation Farming (Figure 4) represent those costs dedicated specifically to conservation farming practices, including the seeds, tools and fertilizers specifically given to lead farmers practicing CF, and to the various trainings received by staff and lead farmers on CF concepts. These accounting costs were separated from the LIG costs as they represent a specific subset of costs incurred for a separate but related project.

Figure 1: Program activity costs in accounting records for Garden set-up cost center

Figure 2: Program activity costs in accounting records for Training & Capacity-building cost center

Figure 3: Program activity costs in accounting records for Garden upkeep/monitoring/follow-up cost center

Figure 4: Program activity costs in accounting records for Conservation Farming cost center
